# Field-deployable visual detection of *Burkholderia gladioli* and its toxigenic pathovar using a one-step RPA-CRISPR/Cas13a assay

**DOI:** 10.1128/spectrum.02143-26

**Published:** 2026-09-25

**Authors:** Jinkai Li, Li Gong, Xiaomeng Huang, Fuming Liu, Jianhua Mei, Siyu Fu, Liyang Qiu, Deyong Zhang, Dongqing Cheng, Xiuying Chen

**Affiliations:** 1 School of Medical Technology, Zhejiang Chinese Medical University, Hangzhou, China; 2 Lishui Center for Disease Control and Prevention, Lishui, China; National University of Singapore, Singapore, Singapore

**Keywords:** *Burkholderia gladioli*, *Burkholderia gladioli *pv. *cocovenenans*, RPA-CRISPR/Cas13a, visual detection, field-deployable

## Abstract

*Burkholderia gladioli* pathovar *cocovenenans* produces the potent mitochondrial toxin bongkrekic acid (BA), causing severe foodborne outbreaks. Conventional detection methods are constrained by prolonged turnaround times and the need for sophisticated instruments, limiting their utility for on-site surveillance. In this study, two independent one-step RPA-CRISPR/Cas13a detection systems were established: one targeting a species-level marker for broad screening of *B. gladioli* and the other targeting the *bonA* gene cluster for specific identification of the toxigenic pathovar. The complete one-step reaction was performed at 37°C for 30 min, and results were visually interpreted using a portable blue-light transilluminator. The visual detection limit reached 1 × 10⁰ copy/μL for both assays, with no cross-reactivity observed against a panel of six non-target strains. Component validation experiments confirmed that all listed constituents were indispensable for fluorescence signal generation. The practical applicability of the platform was evaluated using 100 naturally contaminated black fungus and mushroom substrate samples. The species-level assay returned positive rates of 78% (39/50) for black fungus and 42% (21/50) for substrate samples, while the *bonA*-targeted assay yielded positive rates of 38% (19/50) and 8% (4/50), respectively. The visual readout results were in 100% concordance with conventional quantitative real-time PCR. This field-deployable, equipment-free visual detection platform holds considerable promise for on-site surveillance and early warning of BA-producing *B. gladioli* in food and environmental matrices.

**IMPORTANCE:**

Bongkrekic acid (BA) poisoning caused by *Burkholderia gladioli* pv. *cocovenenans* is a lethal food safety threat, particularly in China and Southeast Asia. Current detection methods depend heavily on laboratory-based instruments, limiting their utility for on-site surveillance. This study developed two independent one-step RPA-CRISPR/Cas13a detection systems: one for broad screening of *B. gladioli* at the species level and the other for specific identification of the toxigenic pathovar. The one-step reaction was completed at 37°C within 30 min, and results were visually interpreted using only a portable blue-light transilluminator. The visual detection limit reached 1 × 10⁰ copy/μL, and the method exhibited 100% concordance with quantitative real-time PCR when applied to 100 naturally contaminated samples. This field-deployable, equipment-free platform provides a practical tool for on-site surveillance and early warning of BA-producing *B. gladioli* in food and environmental matrices.

## INTRODUCTION

*Burkholderia gladioli* pathovar *cocovenenans* (*B. gladioli* pv. *cocovenenans*), a gram-negative bacterium (1), has been identified as the causative agent of severe foodborne outbreaks through the production of bongkrekic acid (BA), a potent mitochondrial toxin (2). The global burden of foodborne illness remains substantial, with the World Health Organization (WHO) estimating approximately 600 million cases and 4.2 million fatalities annually, positioning foodborne diseases among the leading contributors to worldwide morbidity and mortality (3, 4), and BA poisoning represents one of the most lethal forms of foodborne intoxication. The clinical course of intoxication was characterized by acute gastrointestinal symptoms, including vomiting and diarrhea, which rapidly progressed to multiple organ dysfunction syndrome, widespread cellular injury, and death (5). In China, foodborne intoxication caused by this pathogen affected more than 9,000 individuals and resulted in over 1,000 deaths (6). A recent report indicated that nine deaths were recorded in an outbreak in China, with no survivors among the poisoned patients (7).

Given the exceptionally high fatality rate of BA poisoning, effective measures are needed to prevent future outbreaks. A previous study showed that BA biosynthesis was dependent on the presence of the *bon* gene cluster, which directed the assembly of the toxin under natural conditions (8). Consequently, detection of the *bon* cluster was adopted as a molecular indicator for the presence of the toxigenic pathovar (9). In addition to virulence gene detection, the 16S–23S rRNA intergenic spacer region has also been employed as a molecular marker for species-level identification of *B. gladioli* and its differentiation from closely related species (10). Conventional detection methods, including bacterial culture and quantitative real-time PCR (qPCR), were commonly employed for this purpose (11). However, these approaches were often constrained by prolonged turnaround times, heavy reliance on sophisticated laboratory instruments, and considerable operational costs (12).

These limitations have driven the development of alternative detection strategies with improved rapidity, portability, and sensitivity. Over the past decade, emerging technologies based on clustered regularly interspaced short palindromic repeats (CRISPR) and CRISPR-associated (Cas) proteins attracted significant attention for nucleic acid testing owing to their exceptional specificity and sensitivity (13, 14). Originally characterized as an adaptive immune defense mechanism in prokaryotes, CRISPR-Cas systems achieve rapid and sequence-specific recognition and cleavage of target nucleic acids (15, 16). This inherent capacity for sequence-specific targeting has been widely exploited for genetic engineering (17, 18). Among the Cas effectors, Cas13a functioned as an RNA-guided ribonuclease that, upon CRISPR RNA (crRNA)-mediated target recognition, exerted nonspecific collateral cleavage activity against surrounding single-stranded RNA molecules (19, 20). This enzymatic property enabled the conversion of sequence-specific target binding events into measurable signals. Nevertheless, without preamplification, the limit of detection of CRISPR-Cas13a was reported to range between 0.01 and 1 nM, which was insufficient for direct clinical pathogen diagnosis (21). To overcome these limitations, recombinase polymerase amplification (RPA) was integrated as an isothermal nucleic acid amplification step that operated efficiently at 37°C with minimal sample pretreatment (22, 23). The combination of RPA with CRISPR/Cas13a offered a promising strategy for rapid, highly sensitive, and equipment-free on-site pathogen detection (24, 25). In 2017, the specific high-sensitivity enzymatic reporter unlocking (SHERLOCK) platform was established by Gootenberg et al., who successfully coupled Cas13a with RPA to achieve attomolar sensitivity without specialized instrumentation (26).

In this study, two independent RPA-CRISPR/Cas13a detection systems were developed. One system was designed for broad screening of *B. gladioli* at the species level, while the other was tailored for the specific identification of the toxigenic pathovar *B. gladioli* pv. *cocovenenans*. This dual-target strategy provided a reliable technical foundation for on-site prevention and early warning of BA poisoning risk. The established assays were simple to perform, and the detection results could be visually interpreted with a portable blue-light device in field settings, demonstrating their considerable potential for practical application.

## MATERIALS AND METHODS

### Materials and equipment

The RPA amplification kit was sourced from Lesunbio (Wuxi, China). The T7 RNA polymerase, NTPs, ssRNA, crRNA, and LwaCas13a for the CRISPR/Cas13a system were purchased from Sangon Biotech (Shanghai, China). The commercial bacterial DNA extraction kit was obtained from ABT (Beijing, China). Recombinant pUC57 plasmids harboring the species-level marker and the *bonA* gene cluster were constructed and supplied by Sangon Biotech (Shanghai, China). GVC enrichment broth (containing gentian violet and chloramphenicol) was purchased from Huankai Microbiology (Guangdong, China). *B. gladioli* pv. *cocovenenans* ATCC 33664, *B. gladioli* ATCC 10248, *B. cepacia* ATCC 25416, *B. pseudomallei* ATCC 23343, *Pseudomonas aeruginosa* ATCC 27853, *Escherichia coli* ATCC 25922, and *Staphylococcus aureus* ATCC 25923 for specificity detection were provided by the Lishui Municipal Center for Disease Control and Prevention (Lishui, China). Naturally contaminated food and environmental samples, comprising fresh black fungus (*Auricularia auricula-judae*), dried black fungus, and mushroom cultivation substrates collected before and after harvest, were gathered from four major black fungus production zones in Lishui, China. Equipment included an ABI ViiA 7 real-time PCR instrument (Applied Biosystems, USA), a portable blue-light transilluminator (LABGIC, China), and a constant-temperature water bath (Shanghai Yiheng, China).

### DNA extraction

DNA extraction was performed using a bacterial DNA extraction kit according to the manufacturer’s instructions. For pure bacterial cultures used in specificity assays, a single colony (2–3 mm in diameter) was suspended in 200 μL of sterile distilled water, and the suspension was centrifuged at 13,000 rpm for 3 min. The supernatant was discarded, and 50 μL of pre-vortexed nucleic acid extraction buffer was added to the pellet. The mixture was thoroughly resuspended by vortexing, boiled in a water bath for 10 min, and immediately cooled on ice for 3 min. After centrifugation at 13,000 rpm for 10 min, the supernatant was collected and used as the DNA template.

For enriched food and environmental samples, a 500 μL aliquot of GVC enrichment broth was centrifuged at 13,000 rpm for 3 min. The pellet was resuspended in 50 μL of nucleic acid extraction buffer and processed as described above.

### One-step RPA-CRISPR/Cas13a detection

The one-step RPA-CRISPR/Cas13a reaction was assembled in a 20 μL volume containing 10 μL of RPA buffer, 2 μL of NTPs (10 μM), 1.5 μL of nuclease-free water, 1.5 μL of RPA starter reagent, 1 μL of forward primer (10 μM), 1 μL of reverse primer (10 μM), 1 μL of DNA template, 0.5 μL of T7 RNA polymerase, 0.5 μL of LwaCas13a protein (10 μM), 0.5 μL of crRNA (10 μM), and 0.5 μL of ssRNA (10 μM). The sequences of the RPA primers and crRNAs targeting the species-level marker and the *bonA* gene cluster are provided in Table 1.

**TABLE 1:** Primers and crRNAs used in this study

Assay	Oligonucleotide	Sequence (5′−3′)
Species level	Forward primer	CTCAGTCGGTTAGAGCACCGTCTTGATAAGG
	Reverse primer	TAATACGACTCACTATAGGGCAAAGCAGGTGCTCTCCCAGCTGAGCTATG
	crRNA	GAUUUAGACUACCCCAAAAACGAAGGGGACUAAAACACCAATTGTCTGGCGGTAGAAACCTGAG
*BonA* targeted	Forward primer	CTGTTCCTGGAGGAGGGCTACCGCGCCTTC
	Reverse primer	TAATACGACTCACTATAGGGCAGCCAGCCGTATTCGCTCGCGCCCATGAT
	crRNA	GAUUUAGACUACCCCAAAAACGAAGGGGACUAAAACCTACGATCCGCGCCAGCTCAGCGAGGCA

The reaction mixture was incubated at 37°C for 30 min in an ABI ViiA 7 real-time PCR system, with fluorescence signals (FAM channel) recorded at 1 min intervals. Visible fluorescence was observed using a portable blue-light transilluminator.

### Validation of the detection system

To identify the components essential for fluorescence signal generation, a systematic validation experiment was performed for both the species-level and *bonA*-targeted assays. Eight reaction groups (A–H) were established for each assay, each lacking a specific component from the complete one-step RPA-CRISPR/Cas13a system. Group A contained all components, while Groups B–H were each deprived of one component: NTPs, DNA template, T7 RNA polymerase, LwaCas13a protein, crRNA, or ssRNA (Table 2).

**TABLE 2:** Experimental groups for system validation^*a*^

Reagents	A	B	C	D	E	F	G	H
NTPs	+	−	+	+	+	+	+	−
DNA template	+	+	−	+	+	+	+	−
T7 RNA polymerase	+	+	+	−	+	+	+	−
LwaCas13a protein	+	+	+	+	−	+	+	−
crRNA	+	+	+	+	+	−	+	−
ssRNA	+	+	+	+	+	+	−	−

^*a*^ The (+) symbol indicates that the corresponding component was added to the reaction, while the (−) symbol indicates that the component was not added.

### Sensitivity evaluation and limit of detection

To determine the analytical sensitivity of the assay, recombinant plasmids (pUC57) harboring the species-level sequence or the *bonA*-target sequence were constructed. Tenfold serial dilutions of each plasmid were prepared, ranging from 1 × 10³ to 10^0^ copies/μL. Each dilution was tested in triplicate using the established RPA-CRISPR/Cas13a assay.

### Specificity evaluation of the detection system

The specificity of the RPA-CRISPR/Cas13a assay was assessed using genomic DNA extracted from the seven bacterial strains. Each strain was tested in three technical replicates. A no-template control (NTC), in which the DNA template was replaced with nuclease-free water, was included in each run.

### Detection of clinical samples

A total of 100 samples were collected for clinical assessment, comprising 25 fresh black fungus samples, 25 dried black fungus samples, and 25 mushroom substrate samples collected before and after cultivation. All samples were enriched in GVC broth and subjected to DNA extraction as described above. The extracted DNA was subsequently tested using the established RPA-CRISPR/Cas13a assays (both species level and *bonA* targeted) and a conventional qPCR method for comparative accuracy analysis.

### Statistical analysis

For both specificity and sensitivity experiments, fluorescence values obtained from each test group were compared with those of the NTC using one-way analysis of variance followed by Dunnett’s multiple comparison test. A *P* value <0.05 was considered statistically significant. For the purposes of on-site testing, detection results were directly judged by visible fluorescence under a portable blue-light transilluminator, with visible green fluorescence indicating a positive result. All statistical computations and graphical outputs were created with BioGDP.com (27) and generated using GraphPad Prism software version 10.1.2.

## RESULTS

### Establishment of the RPA-CRISPR/Cas13a detection platform

**Fig 1:**
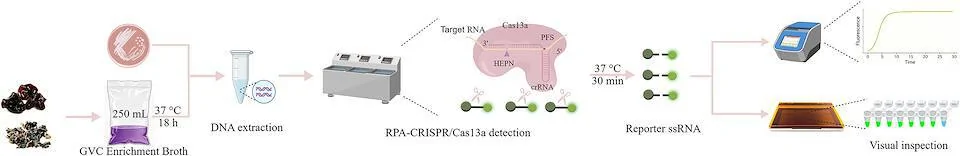
Schematic overview of the one-step RPA-CRISPR/Cas13a detection workflow. The isothermal reaction is completed at 37°C within 30 min. Positive samples emit visible green fluorescence under a portable blue-light transilluminator, while negative samples remain colorless.

The RPA-CRISPR/Cas13a detection workflow was successfully constructed for both the species level and *bonA* targets of *B. gladioli*. Real-time fluorescence detection on the ABI ViiA 7 system revealed typical sigmoidal amplification curves for positive samples, with fluorescence plateauing within 30 min. Positive reactions displayed visible green fluorescence under a portable blue-light transilluminator, whereas negative controls and non-target strains remained colorless (Fig. 1).

### Validation of the RPA-CRISPR/Cas13a detection system

**Fig 2:**
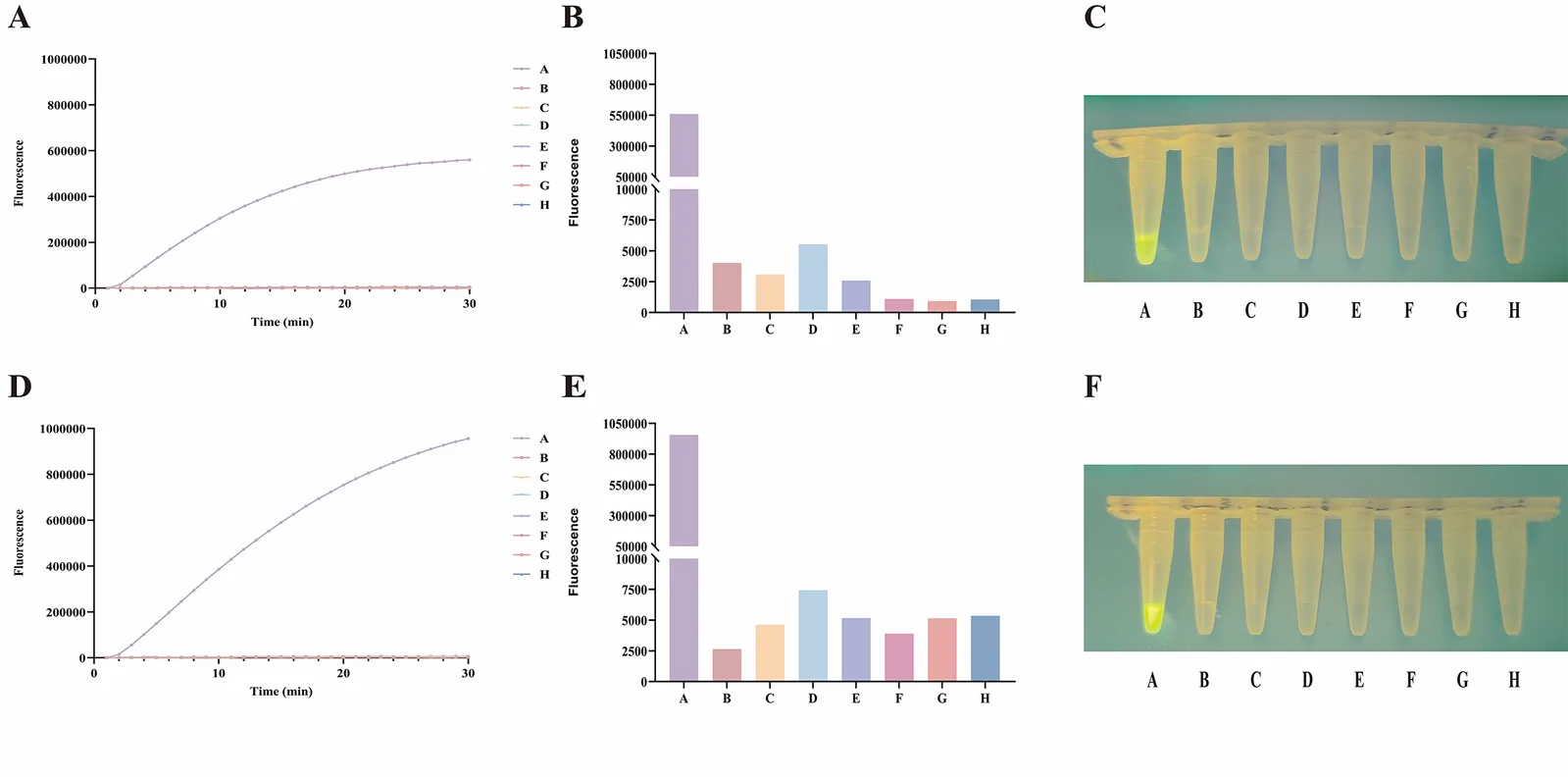
Validation of the one-step RPA-CRISPR/Cas13a system. (**A**) Real-time fluorescence curves and (**B**) endpoint fluorescence values for the species-level assay. (**C**) Visual readout of the species-level assay under a portable blue-light transilluminator. (**D**) Real-time fluorescence curves and (**E**) endpoint fluorescence values for the *bonA*-targeted assay. (**F**) Visual readout of the *bonA*-targeted assay. Group A, complete system; Groups B–H, each lacking one component (see Table 2). Data are presented as mean ± SD from three technical replicates.

The component requirement of the one-step RPA-CRISPR/Cas13a system was evaluated for both the species-level and *bonA*-targeted assays by systematically omitting individual components from the complete reaction (Groups A–H). In both assays, only Group A, which contained all components, produced a strong fluorescence signal; Groups B–H, each deprived of a single constituent, exhibited negligible fluorescence comparable to the NTC (Fig. 2A, B, D, and E). These results confirmed that all listed components were indispensable for signal generation in both detection systems. Consistent with the fluorescence measurements, visible green fluorescence under the portable blue-light transilluminator was observed exclusively in Group A for both assays, whereas all other groups remained colorless (Fig. 2C and F).

### Analytical sensitivity of the RPA-CRISPR/Cas13a assays

**Fig 3:**
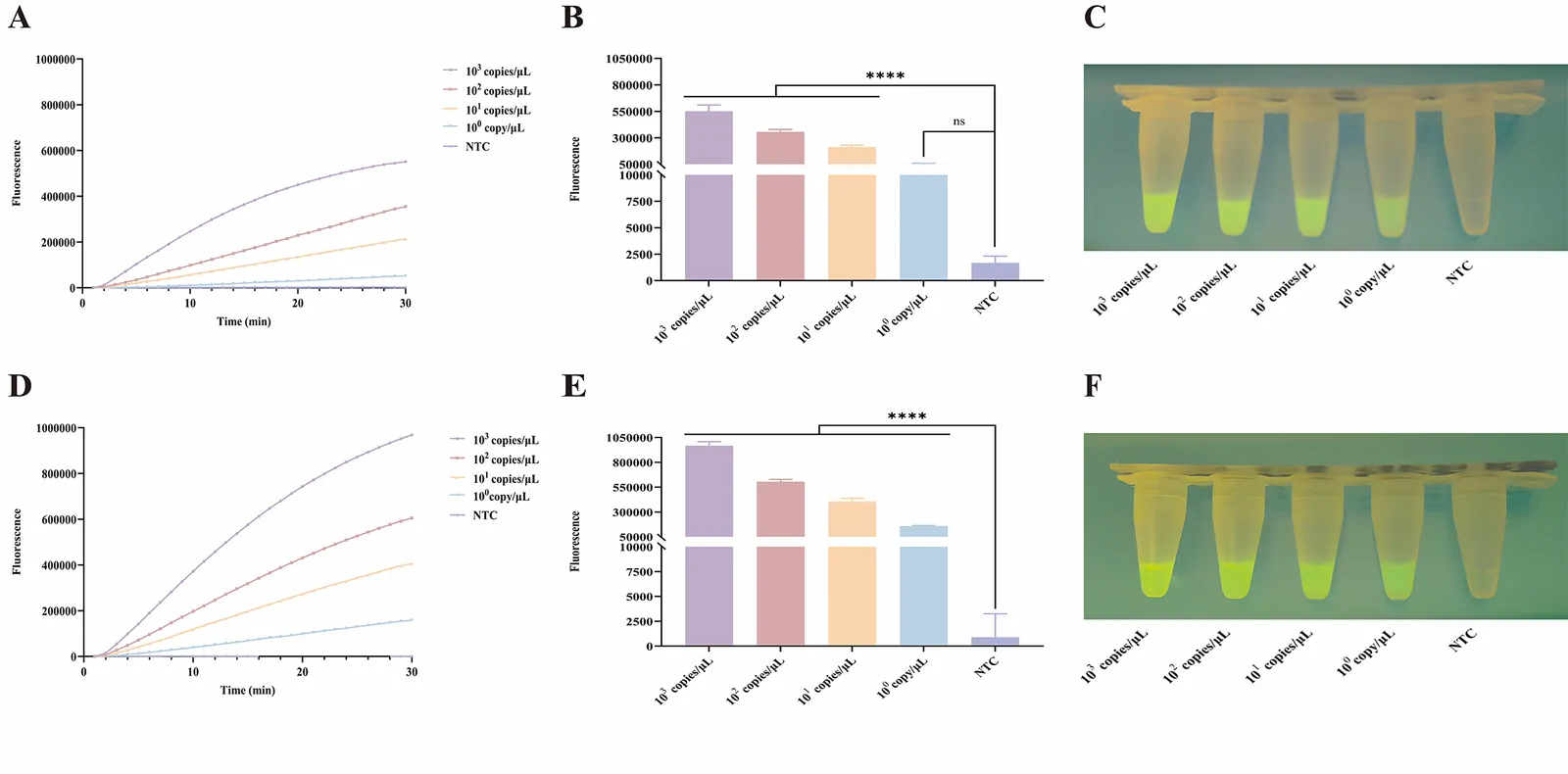
Analytical sensitivity of the RPA-CRISPR/Cas13a assays. (**A**) Real-time fluorescence curves and (**B**) endpoint fluorescence values for the species-level assay using 10-fold serial dilutions of the target plasmid (10³–10^0^ copies/μL). (**C**) Visual readout of the species-level assay under a portable blue-light transilluminator. (**D**) Real-time fluorescence curves and (**E**) endpoint fluorescence values for the *bonA*-targeted assay. (**F**) Visual readout of the *bonA*-targeted assay. The dashed line indicates the mean fluorescence of the NTC plus three standard deviations. *, *P* < 0.05; **, *P* < 0.01; ***, *P* < 0.001; ****, *P* < 0.0001; ns, *P* > 0.05.

The sensitivities of the established RPA-CRISPR/Cas13a assays were evaluated using 10-fold serial dilutions of the recombinant pUC57 plasmids, ranging from 10³ to 10^0^ copies/μL. The species-level assay detected as few as 10^1^ copies/μL of the target plasmid. The endpoint fluorescence intensity at this concentration was significantly greater than that of the NTC (*P* < 0.05) (Fig. 3A and B). The *bonA*-targeted assay exhibited a detection limit of 1 × 10⁰ copy/μL, with a clear distinction from the NTC signal (*P* < 0.05) (Fig. 3D and E). For both assays, fluorescence signals decreased in a concentration-dependent manner. Visible green fluorescence under the portable blue-light transilluminator was observed at plasmid concentrations as low as 1 × 10⁰ copy/μL for the species-level assay and 1 × 10⁰ copy/μL for the *bonA* assay (Fig. 3C and F).

### Specificity of the RPA-CRISPR/Cas13a assays

The specificities of the species-level and *bonA*-targeted RPA-CRISPR/Cas13a assays were separately evaluated using genomic DNA extracted from a panel of seven bacterial strains plus an NTC. Each strain was tested in three technical replicates.

**Fig 4:**
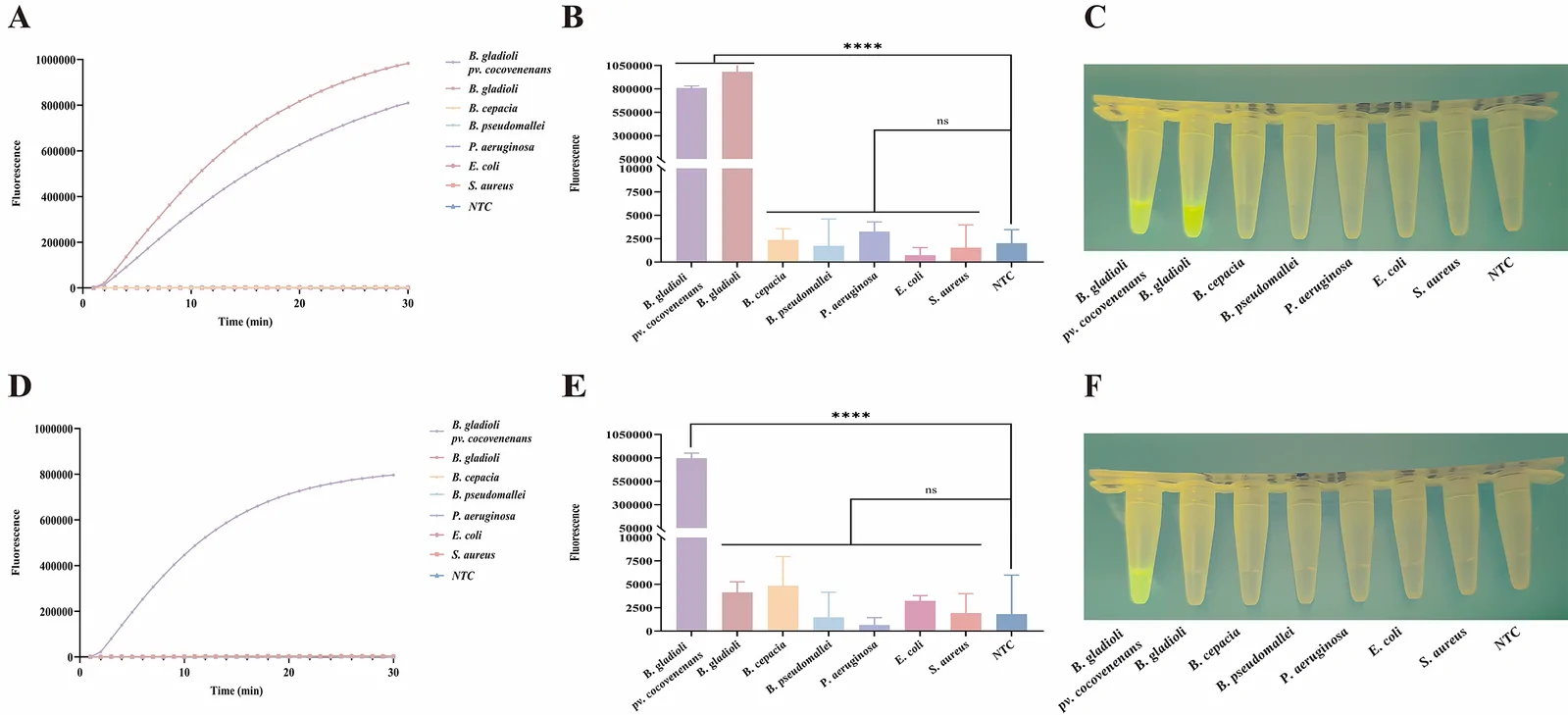
Specificity of the RPA-CRISPR/Cas13a assays. (**A**) Endpoint fluorescence values and (**B**) real-time fluorescence curves for the species-level assay. (**C**) Visual readout of the species-level assay under a portable blue-light transilluminator. (**D**) Endpoint fluorescence values and (**E**) real-time fluorescence curves for the *bonA*-targeted assay. (**F**) Visual readout of the *bonA*-targeted assay. Strains tested: 1, *B. gladioli* pv. *cocovenenans* ATCC 33664; 2, *B. gladioli* ATCC 10248; 3, *B. cepacia* ATCC 25416; 4, *B. pseudomallei* ATCC 23343; 5, *P. aeruginosa* ATCC 27853; 6, *E. coli* ATCC 25922; and 7, *S. aureus* ATCC 25923. *, *P* < 0.05; **, *P* < 0.01; ***, *P* < 0.001; ****, *P* < 0.0001; ns, *P* > 0.05.

For the species-level assay, a strong fluorescence signal was observed exclusively in reactions containing DNA from *B. gladioli* pv. *cocovenenans* ATCC 33664 and *B. gladioli* ATCC 10248 (*P* < 0.05) (Fig. 4A and B), both of which belong to the target species. No cross-reactivity was detected with any of the other five non-target strains. For the *bonA*-targeted assay, fluorescence was detected only in the presence of *B. gladioli* pv. *cocovenenans* ATCC 33664 DNA. All other strains, including *B. gladioli* ATCC 10248, yielded signals comparable to that of the NTC (*P* < 0.05) (Fig. 4D and E). Consistent results were observed under the portable blue-light transilluminator; visible green fluorescence appeared exclusively in the target wells, while all non-target and NTC wells remained colorless (Fig. 4C and F).

### Detection of naturally contaminated samples

To evaluate the practical applicability of the established RPA-CRISPR/Cas13a assays, a total of 100 naturally contaminated samples collected from four major black fungus production areas in Lishui, China, were tested. These samples were divided into two groups: 50 black fungus samples (comprising 25 fresh and 25 dried specimens) and 50 mushroom substrate samples (comprising 25 collected before cultivation and 25 collected after cultivation). Genomic DNA was extracted from each sample following GVC enrichment, and the extracts were subsequently analyzed using the RPA-CRISPR/Cas13a platform with visual readout under a portable blue-light transilluminator and, in parallel, by conventional qPCR.

**Fig 5:**
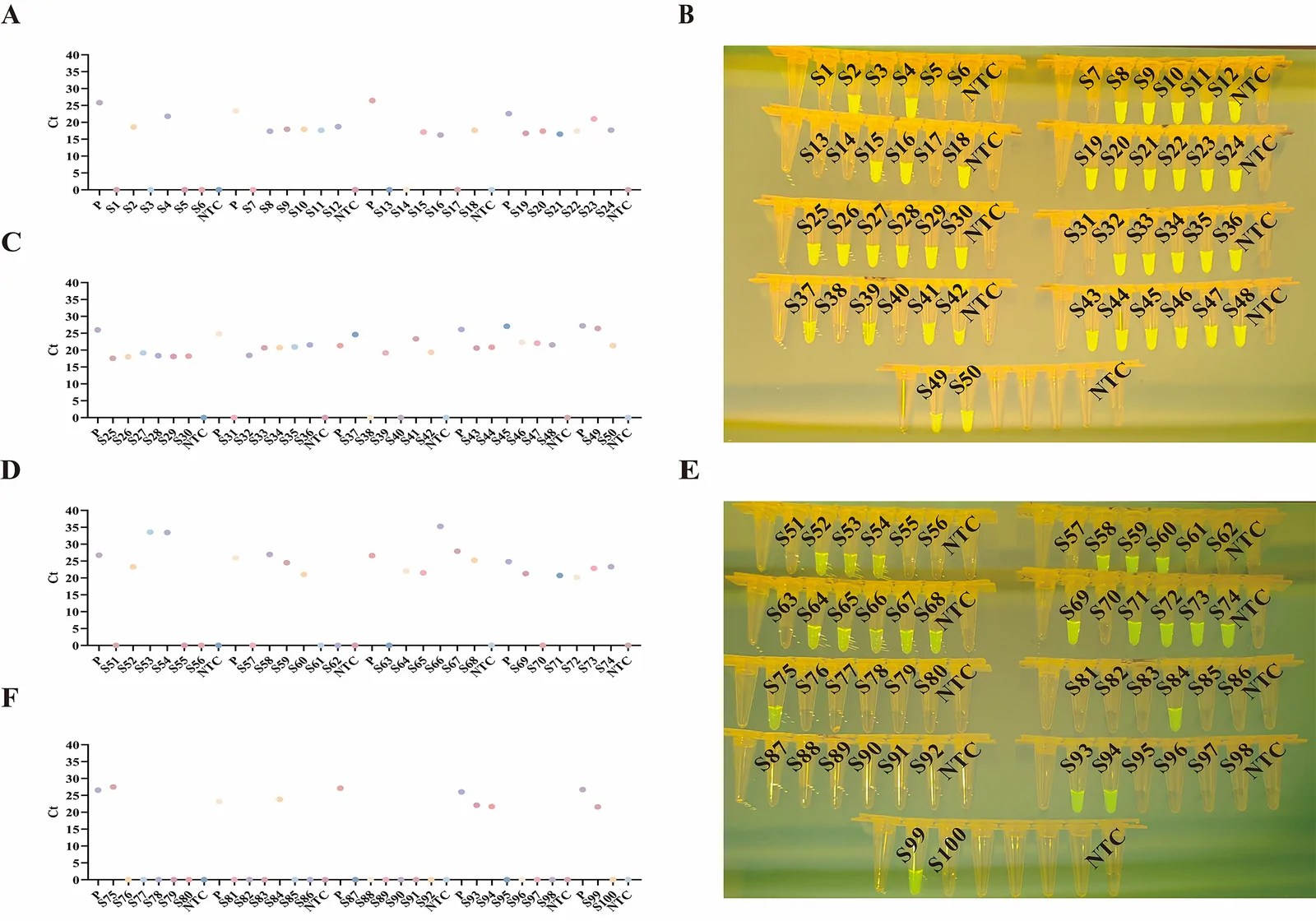
Detection of naturally contaminated black fungus and mushroom substrate samples using the species-level RPA-CRISPR/Cas13a assay. (**A**) qPCR Ct values for samples 1–24. (**C**) qPCR Ct values for samples 25–50. (**D**) qPCR Ct values for samples 51–74. (**F**) qPCR Ct values for samples 75–100. (**B**) Visual readout under a portable blue-light transilluminator for samples 1–50. (**E**) Visual readout for samples 51–100.

**Fig 6:**
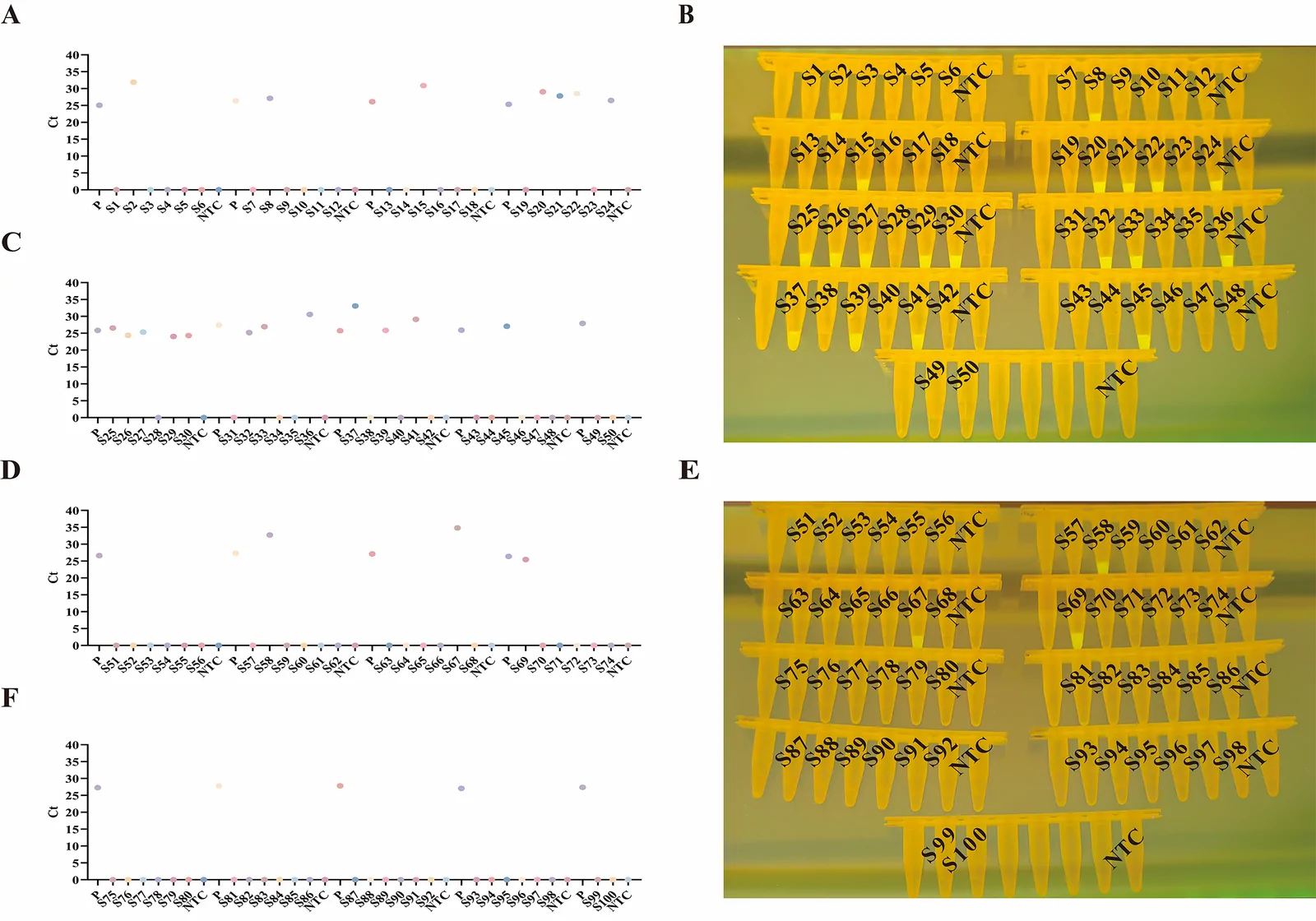
Detection of naturally contaminated black fungus and mushroom substrate samples using the *bonA*-targeted RPA-CRISPR/Cas13a assay. (**A**) qPCR Ct values for samples 1–24. (**C**) qPCR Ct values for samples 25–50. (**D**) qPCR Ct values for samples 51–74. (**F**) qPCR Ct values for samples 75–100. (**B**) Visual readout under a portable blue-light transilluminator for samples 1–50. (**E**) Visual readout for samples 51–100.

Visible green fluorescence was observed under the portable blue-light transilluminator for all positive samples, while negative samples and NTCs remained colorless. Among the 100 samples, the species-level assay returned positive results for 39 of the 50 black fungus samples (78%) and 21 of the 50 mushroom substrate samples (42%) (Fig. 5B and E). The b*onA*-targeted assay, which specifically identifies the toxigenic pathovar, yielded positive results for 19 black fungus samples (38%) and 4 substrate samples (8%) (Fig. 6B and E).

The results obtained by the RPA-CRISPR/Cas13a platform were consistent with those of conventional qPCR (Fig. 5A, C, D, and F, Fig. 6A, C, D, and F). The concordance rate between the two methods was 100% for the species level and 100% for the *bonA* targeted. These results demonstrated that the RPA-CRISPR/Cas13a assays exhibited good diagnostic performance and were suitable for on-site detection of *B. gladioli* and *B. gladioli* pv. *cocovenenans* in food and environmental samples.

## DISCUSSION

BA poisoning, caused by the toxigenic pathovar *Burkholderia gladioli* pv. *cocovenenans*, has remained a persistent food safety concern throughout China and Southeast Asia (28). Conventional detection strategies, reliant on bacterial culture or high-performance liquid chromatography, are constrained by prolonged turnaround times and the requirement for sophisticated laboratory infrastructure (29). Moreover, qPCR assays targeting the *bonA* gene cluster alone cannot discern whether non-toxigenic *B. gladioli* strains are present in a sample—information that holds substantial value for proactive risk assessment. To address these limitations, two independent RPA-CRISPR/Cas13a detection modules were established in this study: a species-level screening assay for broad identification of *B. gladioli* and a *bonA*-targeted assay for the exclusive detection of the BA-producing pathovar.

The visual sensitivity of the platform was evaluated by direct observation of fluorescence under a portable blue-light transilluminator using 10-fold serial dilutions of recombinant plasmids. Visible green fluorescence was unambiguously observed at concentrations as low as 1 × 10⁰ copy/μL for both the species-level and *bonA*-targeted assays. These visual detection limits were consistent with the statistically significant fluorescence signals recorded by the qPCR instrument, confirming that naked-eye interpretation under blue light can achieve sensitivity comparable to instrumental readout. Given that naturally contaminated samples following GVC enrichment typically harbor bacterial loads substantially exceeding these levels, the visual sensitivity of both assays was considered adequate for field-based screening applications.

The visual specificity of the platform was assessed against a panel of seven bacterial strains. Under the portable blue-light transilluminator, green fluorescence appeared exclusively in the target wells, while all non-target strains and no-template controls remained completely colorless. Specifically, the species-level assay produced visible fluorescence only for the two *B. gladioli* strains—*B*. *gladioli* pv. *cocovenenans* and *B. gladioli*—with no visible signal from *B. cepacia*, *B. pseudomallei*, *P. aeruginosa*, *E. coli*, or *S. aureus*. The *bonA*-targeted assay yielded visible fluorescence solely for the toxigenic strain; all other strains, including the closely related *B. gladioli*, remained indistinguishable from the NTC. These visual results were in full agreement with the fluorescence intensity measurements obtained instrumentally, and the binary nature of the readout—fluorescence either clearly visible or completely absent—eliminated any ambiguity in result interpretation. This divergent visual reactivity pattern between the two assays confirms their intended design: one enables broad species-level screening by naked eye, while the other permits pathovar-level identification without instrumentation.

The dual-target architecture of this platform carries practical epidemiological implications. A sample testing positive by the species-level assay but negative by the *bonA* assay signals the presence of non-toxigenic *B. gladioli*—an indication that the sampled environment already supports colonization by this bacterial species. Mounting genomic evidence suggests that the *bon* gene cluster resides on mobile genetic elements, raising legitimate concerns that non-toxigenic strains could acquire the toxigenic capacity through horizontal gene transfer (30). Species-level monitoring could therefore serve as an early warning system, flagging high-risk habitats before toxigenic variants emerge and contaminated products enter the food supply chain.

The field applicability of the platform was validated by analyzing 100 naturally contaminated samples collected from four black fungus production areas in Lishui, China. Among the 50 black fungus samples, the species-level assay returned positive results for 39 samples (78%), while the *bonA*-targeted assay identified the toxigenic pathovar in 19 samples (38%). Among the 50 mushroom substrate samples, the species-level assay yielded 21 positive results (42%), with the *bonA* assay detecting the toxigenic strain in only 4 samples (8%). The higher detection rate in black fungus compared with cultivation substrates suggests that the edible fungus itself, rather than the growth medium, may serve as the primary reservoir for *B. gladioli* contamination. Notably, the RPA-CRISPR/Cas13a results were in complete agreement with conventional qPCR findings, achieving 100% concordance for both the species level and *bonA* targeted. This perfect agreement demonstrates that the developed platform offers diagnostic accuracy comparable to the current gold standard molecular method, while providing substantial advantages in speed and portability. All positive samples were unambiguously identified by visual inspection under a compact blue-light transilluminator, eliminating the requirement for real-time fluorescence monitoring equipment and underscoring the platform’s suitability for point-of-need applications, including farm-side inspections, food processing audits, and border quarantine screening.

Several methodological aspects of the current approach merit discussion. The entire analytical workflow, from enriched culture to visual readout, was completed within approximately 19 h—substantially faster than the multi-day turnaround of culture-based methods and competitive with qPCR protocols when sample transport and batch processing are considered. The isothermal nature of RPA obviates the need for thermal cycling equipment, while the crRNA-directed collateral cleavage activity of Cas13a provides an additional layer of sequence-specific validation that mitigates the risk of false-positive results associated with nonspecific amplification (31, 32). Compared with other isothermal techniques, the RPA-CRISPR/Cas13a system benefits from straightforward primer design and a lower operating temperature, both of which enhance its field compatibility.

Several limitations of this study should be acknowledged. First, although the sample size (*n* = 100) was adequate for an initial performance assessment, the geographic scope was limited to four production zones within a single region of China. Broader multi-site validation encompassing diverse climatic conditions, soil types, and agricultural practices would strengthen confidence in the generalizability of the assay. Third, it should be emphasized that the *bonA* assay detects the genetic potential for BA biosynthesis rather than the toxin itself; the quantitative relationship between *bonA* gene abundance and actual BA concentration under varying environmental parameters warrants further investigation.

In conclusion, this study established a rapid, specific, and visually interpretable RPA-CRISPR/Cas13a platform for dual-level detection of *B. gladioli*—integrating broad species coverage with precise toxigenic pathovar identification. The platform achieved detection limits of 1 × 10⁰ copy/μL for the species level and *bonA* targeted, respectively, with 100% specificity against non-target organisms. On 100 naturally contaminated black fungus and substrate samples, the platform demonstrated 100% concordance with qPCR, confirming its diagnostic reliability. The straightforward visual readout format makes this methodology a practical candidate for on-site food safety surveillance, contributing to the prevention and early warning of BA poisoning.

## Data Availability

All data supporting the findings of this study are available within the article. Additional raw data are available from the corresponding author upon reasonable request.
